# POSITIVE EMOTIONAL CONNECTION AND CAREGIVER WELL-BEING IN BEHAVIORAL-VARIANT FRONTOTEMPORAL DEMENTIA

**DOI:** 10.1093/geroni/igac059.1577

**Published:** 2022-12-20

**Authors:** Kuan-Hua Chen, Claire Yee, Casey Brown, Anna Sapozhnikova, Peter Pressman, Jennifer Merrilees, Barbara Fredrickson, Robert Levenson

**Affiliations:** University of California, Berkeley, Berkeley, California, United States; University of California, Berkeley, Berkeley, California, United States; Georgetown University, Washington, District of Columbia, United States; Pacific Anxiety Group, San Francisco, California, United States; University of Colorado School of Medicine, Aurora, Colorado, United States; University of California, San Francisco, San Francisco, California, United States; University of North Carolina at Chapel Hill, Chapel Hill, North Carolina, United States; University of California, Berkeley, Berkeley, California, United States

## Abstract

Behavioral-variant frontotemporal dementia (bvFTD) is characterized by impairment in socioemotional functioning. Spouses caring for individuals with bvFTD often experience profound health/well-being declines, compared to Alzheimer’s disease (AD) caregivers and non-caregiving older adults. We hypothesized that disrupted positive emotional connections between spousal caregivers and individuals with bvFTD contribute to caregivers’ lower emotional well-being. 23 bvFTD-caregiver, 23 AD-caregiver, and 17 control dyads had a 10-minute conflict conversation in the laboratory. Positive emotional connections were measured as the covariation of partners’ positive emotional behaviors during the conversation. Caregiver emotional well-being was assessed via questionnaire (SF-36). We found that bvFTD caregivers had lower emotional well-being than AD caregivers and controls (who did not differ from each other, t=.80, p=.43), c=-.70, p<.01. Importantly, this effect was fully mediated by bvFTD caregivers' lower positive emotional connections, c’=-.38, n.s. We speculate that lower positive emotional connections can cause social isolation and contribute to bvFTD caregivers’ health/well-being declines.

